# Perceived Stress and Compassion Satisfaction Among Nurses: A Mediation Model of Perceived Disaster Preparedness

**DOI:** 10.1097/jnr.0000000000000729

**Published:** 2026-02-11

**Authors:** Kai-Jo CHIANG, Li-Fang CHANG, Lin-Ju CHOU, Li-Chun HUANG, Yu-Ling KAO, Chin LIN, Ya-Hung CHEN, Ai-Hsiu HUNG, Hsueh-Hsing PAN

**Affiliations:** 1 Department of Nursing, Tri-Service General Hospital, Taipei, Taiwan, ROC; 2 College of Nursing, National Defense Medical University, Taipei, Taiwan, ROC; 3 Department of Medical Education, Taipei Medical University Hospital, Taiwan, ROC; 4 School of Nursing, College of Nursing, Taipei Medical University, Taipei, Taiwan, ROC; 5 Graduate Institute of Medical Sciences, College of Medicine, National Defense Medical University, Taiwan, ROC; 6 Department of Nursing, Kaohsiung Armed Forces General Hospital, Kaohsiung, Taiwan, ROC; 7 Office of Medical Education and Research, Zuoying Armed Forces General Hospital, Kaohsiung, Taiwan, ROC; 8 School of Nursing, Fooyin University, Kaohsiung, Taiwan, ROC; 9 Medical Technology Education Center, School of Medicine, College of Medicine, National Defense Medical University, Taipei, Taiwan, ROC; 10 Military Digital Medical Center, Tri-Service General Hospital, National Defense Medical University, Taipei, Taiwan, ROC; 11 Department of Nursing, Taichung Armed Forces General Hospital, Taiwan, ROC; 12 Department of Nursing, Hualian Armed Forces General Hospital, Taiwan, ROC; † Contributed equally

**Keywords:** compassion satisfaction, perceived stress, perceived disaster preparedness, nurses

## Abstract

**Background:**

The tremendous stress that nurses experience during disaster events can affect their compassion satisfaction (CS) at work. Despite the recognized negative effects of perceived stress (PS) on perceived disaster preparedness (PDP) and CS, little is known regarding the mediating role of PDP on the PS–CS relationship.

**Purpose:**

This study was conducted to explore the significance of the PS effect on CS through PDP in hospital nurses.

**Methods:**

A cross-sectional study design was used. Nine hundred and seventy-three nurses from hospitals in Taiwan participated in this study, which was implemented between July and October 2020. Data were collected using a basic characteristics datasheet, the Perceived Stress Scale (PSS-14), and the Compassion Satisfaction Scale (CSS). A mediation model was used to test the mediating effect of PDP on the relationship between PS and CS after adjusting for covariates, and stratified analysis was conducted to explore the degree of this indirect effect within the subgroups defined by each variable.

**Results:**

PDP was found to significantly mediate the relationship between PS and CS in the sample (indirect effect: −0.104, 95% confidence interval: [−0.12, −0.08]). Using stratified analysis, a significant indirect effect in >30% of participants was found in the following three subgroups: >40 years old (indirect effect=−0.168, *p*=.016), working at hospitals in southern Taiwan (indirect effect=−0.138, *p*=.018), and working day and night shifts (indirect effect=−0.126, *p*=.023).

**Conclusions/Implications for Practice:**

The findings support PDP as a resilience factor that mitigates the impact of PS on CS in hospital nurses. To enhance CS, targeted interventions such as training programs should be implemented to reduce PS and strengthen PDP, particularly in higher-risk subgroups of nurses.

## Introduction

Disasters are severe events attributable to either human-caused or natural hazards that disrupt normal conditions and inflict significant suffering exceeding the capacity of the affected community or society to manage using their own resources (X. Mao et al., 2022). The COVID-19 pandemic is a disaster that originated in China at the end of 2019 and spread globally in 2020 (World Health Organization, 2024). Taiwan has recorded over 15 million cases of COVID-19 in the country and ~5 million confirmed cases and nearly 10,000 deaths since 2020 (Taiwan National Infectious Disease Statistics System, 2023).

Taiwan’s health care system, supported by a well-established National Health Insurance program, is renowned for its efficiency and accessibility. However, the country’s geographic location and high population density make it particularly vulnerable to natural disasters such as typhoons, earthquakes, and floods, as well as public health crises like COVID-19. Taiwan’s disaster response system emphasizes a coordinated, multisectoral approach that includes strict government policies, community-based interventions, and rapid health care mobilization (Bosner & Chang, 2020).

During disasters, nurses play a critical role as frontline responders, fighting to protect lives under immensely stressful conditions. Hospital nurses, in particular, face considerable challenges, including responding rapidly to government policy priorities, making sound clinical judgments, and providing comprehensive support when confronting a high volume of casualties and critical events (Luo et al., 2023). Consequently, hospital nurses often face significant challenges that may subsequently impact their compassion satisfaction (CS; Lu et al., 2020).

As a component of professional quality of life, CS represents a positive emotion that arises from a sense of self-satisfaction derived from assisting others (Stamm, 2010). Among health care professionals, CS is a sense of fulfillment that arises from providing care for their suffering patients. This sense of fulfillment has the potential to impact patient-care quality, safety, and outcomes while also contributing to greater well-being and job satisfaction (Okoli et al., 2020). Perceived stress (PS) refers to an individual’s subjective feelings or personal assessment of stressful events (Cohen et al., 1983). A negative correlation between PS and CS has been reported in numerous previous studies (e.g., Maddigan et al., 2023; Shi et al., 2023; Zakeri et al., 2021).

Perceived disaster preparedness (PDP) encompasses an individual or group’s subjective evaluation of their readiness and capacity to cope effectively with potential disasters or emergency situations. This assessment includes factors such as knowledge, skills, resources, and the competencies to respond adeptly in the face of such circumstances (Abu Hasheesh, 2023; Choi et al., 2022). PDP plays a crucial role in enhancing nurses’ confidence, sense of security, and behaviors in the face of disaster-related challenges. A recent study on disaster nursing found that nurses with greater disaster management competency and motivation tended to report lower stress levels (Kula Sahin & Aydin, 2024). Furthermore, the study highlighted that factors such as age, educational level, professional experience, disaster nursing training, and knowledge of disaster response roles significantly influence PDP. These findings suggest PDP is shaped by both individual characteristics and professional training, reinforcing its potential role in buffering stress and enhancing well-being.

Empirical evidence further supports the role of PDP in mitigating stress. A cross-sectional study involving health care workers found higher PDP to correlate positively with lower PS and greater job satisfaction (Afulani et al., 2021). Moreover, prior studies have demonstrated PDP as negatively correlated with CS and as a predictor of CS in nurses (Jang & Cho, 2022; Zakeri et al., 2021). These findings underscore the possibility that PDP may function as a protective factor that enhances the ability of nurses to cope with stress and maintain professional fulfillment.

Although the direct relationships between PS, CS, and PDP have been explored in the literature, the potential mediating role of PDP remains underexamined, particularly in the context of hospital nurses. Notably, not all nurses experience the same decline in CS when exposed to high stress, suggesting that PDP may account for individual differences in psychological response to disaster events. Given that PDP reflects disaster-specific competencies and psychological preparedness, it is reasonable to hypothesize that higher PDP enables nurses to better manage stress, thereby attenuating the negative impact of PS on CS.

Given these theoretical and empirical foundations, PDP is hypothesized in this study to mediate the relationship between PS and CS. This mediation model is based on the premise that nurses with higher PDP are better equipped to handle stress, which may, in turn, foster greater CS. Understanding this relationship is critical, as identifying protective psychological factors such as PDP can inform targeted interventions aimed at supporting nurses’ well-being in high-stress environments. To address these research gaps, this study was designed to examine whether PDP mediates the PS and CS relationship in hospital nurses. The hypotheses of this study were:

Hypothesis 1: PS is negatively associated with CS.

Hypothesis 2: PDP is positively associated with CS.

Hypothesis 3: PDP mediates the relationship between PS and CS.

## Methods

### Study Design and Participants

In this study, a cross-sectional and descriptive approach was employed to investigate the potential mediating role of PDP in the relationship between PS and CS among hospital nurses in Taiwan. The participants were recruited in equal proportions from hospitals located in northern, central, southern, and eastern Taiwan during the period from July to October 2020. The inclusion criteria specified that participants should be registered nurses with experience working in hospitals who were willing to participate. Otherwise eligible nurses who were not employed in hospitals, declined to provide consent, or did not complete the questionnaire were excluded from the sample. Of the 1,001 nurses enrolled as participants, 973 returned valid questionnaires and their data were included in the analysis, resulting in a completion rate of 97.2%.

### Instruments

The questionnaire used in this study included a demographic and work-related characteristics datasheet, the PSS-14, PDP measurement, and the CS scale.

### Demographic and Work-Related Characteristics

Data on several demographic factors, including age, gender, educational level, religious beliefs, marital status, parental status, living arrangements, and level of family support, were collected from the participants. The level of family support was classified as either “very high” or “other.” Also, data on several work-related characteristics, including region of hospital (northern, central, southern, or eastern Taiwan), ward (general ward, intensive care unit [ICU], or emergency room), length of nursing service, job position (either “not in leadership” or “assistant head nurse or above”), shift status (day shift, night shift, or rotating shifts), participation in stress-related education, and involvement in disaster nursing training.

### Perceived Stress Scale (PSS-14)

The PSS-14 is widely utilized to assess PS level in individuals (Cohen et al., 1983). Its Taiwanese translation has demonstrated satisfactory validity and reliability (Chu & Kao, 2005). The scale comprises 14 items, evenly divided into 7 positive and 7 negative statements. Respondents rate each item on a scale from 0 to 4, with 0=“*never*” and 4=“*always*.” In calculating the total score, positive item scores are converted, resulting in a possible scale score range of 0 to 56, with higher scores indicating a higher PS level. In this study, the Cronbach α reliability coefficient for the PSS-14 scale was .848.

### Perceived Disaster Preparedness (PDP) level

The PDP scale is a single-item, self-reported measure developed by the author to assess the subjective perception of the participants regarding their preparedness for responding to a disaster event. It consists of the question, “How would you rate your perceived level of preparedness in responding to a disaster event?” and is rated on a 5-point Likert scale, with 1 indicating *very low*, 2 indicating *low*, 3 indicating *medium*, 4 indicating *high*, and 5 indicating *very high preparedness* levels. As a single-item measure, the internal consistency reliability (e.g., Cronbach α) of this measure is not applicable. However, to ensure validity, the scale was reviewed by experts in disaster preparedness, who confirmed its content validity.

### Compassion Satisfaction Scale (CSS)

The CSS is a component of the Professional Quality of Life tool developed by Stamm (2009). The CSS has previously demonstrated sound construct validity and reliability, with a Cronbach α of .71. The CSS comprises 10 items, each scored on a scale of 1 to 5, with 1 corresponding to “*never*” and 5 corresponding to “*always*.” In calculating total scores, the values of reverse items are adjusted. The total possible scale score ranges from 10 to 50 points, with higher scores indicating higher levels of CS. Based on quartiles, CSS scores are classified into three categories: low (below 25%), moderate (25%–75%), and high (above 75%). In this study, the Cronbach α reliability coefficient for the CSS was .929.

### Study Procedure

This research received ethical approval from the institutional review board (IRB) of both the medical center (B202005010) and Kaohsiung Armed Forces General Hospital (KAFGHIRB 109-048). Subsequent to obtaining IRB approval, the project investigator initiated contact with the Director of the Nursing Department at each hospital. The objective was to recruit ~1,000 nurses, with equal representation from each hospital. The project investigator provided a comprehensive explanation of the purpose and methodology of the study to each Nursing Department director, and nurses who met the inclusion criteria were invited to participate and asked to sign written informed consent. Data collection used the above-described questionnaire, which took ~15 minutes to complete. Confidentiality of the data was ensured, and participants had the right to withdraw from the study at any time.

### Statistical Analysis

The gathered data were organized and stored using Microsoft Excel, while the analysis was conducted using IBM SPSS Statistics for Windows 20.0 (IBM Corp., Armonk, NY, USA). For continuous variables, descriptive statistics such as mean and standard deviation (*SD*) were utilized, while categorical variables were described using frequency and percentage. To explore the relationships between PS, PDP, and CS, bivariate correlations were employed. A level of *p*<.05 was used throughout to determine statistical significance.

Bonferroni correction was applied to address the multiple testing in the examination of bivariate correlations among the study variables, and a series of multiple regression analyses were conducted to assess the mediation effects of PDP on the relationship between PS and CS. These analyses were used to evaluate the associations between PS and PDP (*a* path), PDP and CS (*b* path), and PS and CS (*c* path, also referred to as “total effects”), while controlling for covariates. Furthermore, the direct effects of PS on CS were computed considering PDP and the other covariates (*c* *′* path). To determine the significance of the mediation effects, defined by the product of the regression estimates for the *a* and *b* paths, a bootstrapping method with 1,000 bootstrapped samples (ref) was employed. A mediation effect was considered significant when the 95% bias-corrected bootstrap confidence interval (CI) did not include 0. In addition, stratified analysis was utilized to investigate the proportion of the indirect effect of PDP on the association between PS and CS within the subgroups defined by each variable.

## Results

### Demographic and Work-Related Characteristics

The average age of participants was 33.3 years. Nearly half were under 30 years old (45.6%) and a majority were female (92.8%), single (61.4%), and religious (56.2%); held a bachelor’s degree (69.9%); had no children (66.8%), and lived with others (80.7%). In terms of support, 40.3% reported receiving a high level of support, with the remainder (59.7%) reporting other levels. In terms of work-related characteristics, half of the participants worked in hospitals in northern Taiwan (50.8%), and most were employed in either a general ward (42.4%) or the ICU (42.9%). Most were not in leadership positions (84.5%). Slightly less than half (44.2%) worked night shifts only, 56.4% had not participated in stress-related education, and 51.3% had prior disaster nursing training experience. The mean number of years of service was 10 years (Table 1).

**Table 1:** Participant Demographic and Work-Related Characteristics (*N*=973)

Variable	*n* (%)
**Demographics**	
Age (years; mean and *SD*)	33.3 (9.2)
<30	444 (45.6)
30–40	316 (32.5)
>40	213 (21.9)
Gender	
Female	903 (92.8)
Male	70 (7.2)
Educational level	
Junior college	159 (16.3)
Bachelor	680 (69.9)
Master	134 (13.8)
Religious beliefs	
No	426 (43.8)
Yes	547 (56.2)
Marital status	
Married	376 (38.6)
Single	597 (61.4)
Children	
No	650 (66.8)
Yes	323 (33.2)
Living with others	
No	188 (19.3)
Yes	785 (80.7)
Family support (*M* and *SD*)	4.2±0.8
Very high	392 (40.3)
Other level	581 (59.7)
**Work-related characteristics**	
Geographic location	
Northern	494 (50.8)
Central	116 (11.9)
Southern	297 (30.5)
Eastern	66 (6.8)
Ward	
General ward	413 (42.4)
Intensive care unit	417 (42.9)
Emergency room	143 (14.7)
Length of nursing service (years; *M* and *SD*)	10.0±9.9
Position	
Not in leadership positions	822 (84.5)
Assistant head nurse or above	151 (15.5)
Shift schedule	
Day shift only	208 (21.4)
Night shift only	430 (44.2)
Rotating shift	335 (34.4)
Stress-related education experience	
Yes	424 (43.6)
No	549 (56.4)
Disaster nursing training experience	
No	474 (48.7)
Yes	499 (51.3)

### Descriptive Statistics and Bivariate Correlations Among PS, PDP, and CS

The mean scores for PS and PDP were 24.5 (*SD*=7.3) and 3.4 (*SD*=0.7), respectively, while the mean score for CS was 33.2 (*SD*=7.1). Most of the participants demonstrated a moderate level of CS, accounting for 47.3% of the sample. The details are shown in Table 2.

**Table 2:** Perceived Stress, Perceived Disaster Preparedness, and Compassion Satisfaction Levels (*N*=973)

Variable	Mean (*SD)**/**n* (%)
Perceived stress	24.5±7.3
Perceived disaster preparedness	3.4±0.7
Compassion satisfaction	33.2±7.1
Low	280 (28.8)
Moderate	460 (47.3)
High	233 (23.9)

The bivariate correlations among PS, PDP, and CS, displayed in Table 3, show significant associations between higher levels of PS and lower levels of, respectively, PDP (*r*=−.36, *p*=.019) and CS (*r*=−.42, *p*<.001). Moreover, higher levels of PDP were found to be significantly associated with higher levels of CS (*r*=.46, *p*<.001).

**Table 3:** Bivariate Correlation Among Perceived Stress, Perceived Disaster Preparedness, and Compassion Satisfaction (*N*=973)

Variable	Perceived Stress	Perceived Disaster Preparedness *r* (*p*)	Compassion Satisfaction *r* (*p*)
Perceived stress	1	−.36 (.019)	−.42 (<.001)
Perceived disaster preparedness		1	.46 (<.001)
Compassion satisfaction			1

### Analyses of PDP as a Mediator in the Relationship Between PS and CS

**Figure 1:**
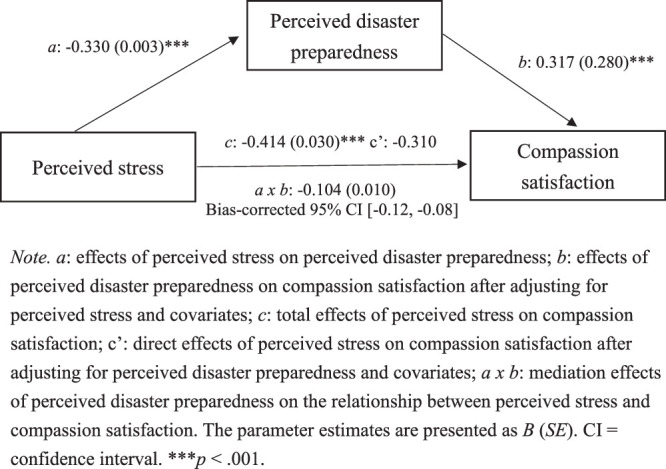
Mediation Effects of Perceived Disaster Preparedness (PDP) on the Relationship Between Perceived Stress (PP) and Compassion Satisfaction (CS)

The results of the analyses on whether PDP acts as a mediator between PS and CS are shown in Figure 1. As expected, the total effects of PS on CS were found to be significant (*c* path: *B*=−0.414, *p*<.001). Furthermore, these effects were mediated by PDP (*a × b: B*=−0.104, 95% bias-corrected bootstrap CI: [−0.12, −0.08]), indicating that, in nurses, the impact of PS on CS comes mainly through PDP. Even after accounting for PDP and other covariates, the effects of PS on CS remained significant (*c* *′* path: *B*=−0.310, *p*<.001), suggesting a partial mediation. In addition, the effects from PS to PDP (*a* path: *B*=−0.330, *p*<.001) and from PDP to CS (*b* path: *B*=0.317, *p*<.001) were also significant after controlling for PS and the other covariates.

### Stratified Analysis of Path Modeling for PS, PDP, and CS

Stratified analysis was used to explore the proportion of the indirect effect of PDP on the relationship between PS and CS within the subgroups defined by each variable. The results reveal that the proportion of the indirect effect, exceeding 30%, was statistically significant in the following three subgroups: participants older than 40 years (indirect effect=−0.168, *p*=.016, accounting for 36% of indirect effect); those working in hospitals located in southern Taiwan (indirect effect=−0.138, *p*=.018, accounting for 35% of indirect effect); and those working a rotating (day and night) shift schedule (indirect effect=−0.126, *p*=.023, accounting for 37% of indirect effect). These findings emphasize the critical role, and especially among these subgroups, of PDP as a mediator in the relationship between PS and CS in nurses. Further details are presented in Table 4.

**Table 4:** Stratified Analysis of Path Modeling for Perceived Stress, Perceived Disaster Preparedness, and Compassion Satisfaction (*N*=973)

Variable	PS→CS		PS→CS		PS→PDP→CS		
	Crude Effect	*p*	Direct Effect	*p*	Crude Effect	*p*	Proportion to Indirect Effect (%)
**Demographics**							
Age							
<30 years	−0.394	<.001	−0.306	<.001	−0.088	.066	22
30–40 years	−0.399	<.001	−0.331	<.001	−0.068	.234	17
>40 years	−0.467	<.001	−0.300	<.001	−0.168	.016	36
Gender							
Female	−0.399	<.001	−0.331	<.001	−0.068	.001	17
Male	−0.383	.001	−0.289	.012	−0.094	.439	25
Educational level							
Junior college	−0.318	<.001	−0.200	.011	−0.118	.141	37
Bachelor	−0.394	<.001	−0.301	<.001	−0.093	.016	24
Master	−0.582	<.001	−0.469	<.001	−0.113	.197	19
Religious beliefs							
No	−0.446	<.001	−0.340	<.001	−0.106	.030	24
Yes	−0.389	<.001	−0.285	<.001	−0.105	.015	27
Marital status							
Married	−0.420	<.001	−0.310	<.001	−0.109	.035	26
Single	−0.408	<.001	−0.314	<.001	−0.094	.023	23
Children							
No	−0.412	<.001	−0.316	<.001	−0.096	.015	23
Yes	−0.416	<.001	−0.308	<.001	−0.108	.054	26
Living with others							
No	−0.400	<.001	−0.290	<.001	−0.109	.137	27
Yes	−0.417	<.001	−0.313	<.001	−0.104	.004	25
Family support							
Very high support	−0.361	<.001	−0.257	<.001	−0.103	.042	25
Other level	−0.401	<.001	−0.330	<.001	−0.071	.087	13
**Work-related characteristics**							
Geographic location							
Northern	−0.461	<.001	−0.356	<.001	−0.106	.019	23
Central	−0.221	.017	−0.173	.063	−0.049	.603	22
Southern	−0.397	<.001	−0.259	<.001	−0.138	.018	35
Eastern	−0.528	<.001	−0.478	<.001	−0.049	.695	9
Ward							
General ward	−0.446	<.001	−0.314	<.001	−0.131	.008	29
Intensive care unit	−0.406	<.001	−0.306	<.001	−0.100	.042	25
Emergency room	−0.332	<.001	−0.262	<.001	−0.070	.409	21
Position							
Not in a leadership position	−0.364	<.001	−0.265	<.001	−0.098	.005	27
Assistant head nurse or above	−0.681	<.001	−0.611	<.001	−0.069	.398	10
Shift schedule							
Day shift only	−0.601	<.001	−0.479	<.001	−0.123	.080	20
Night shift only	−0.344	<.001	−0.289	<.001	−0.055	.253	16
Rotating shift	−0.345	<.001	−0.219	<.001	−0.126	.023	37
Stress-related education experience							
Yes	−0.472	<.001	−0.364	<.001	−0.108	.027	23
No	−0.373	<.001	−0.277	<.001	−0.096	.025	26
Disaster nursing training experience							
No	−0.462	<.001	−0.352	<.001	−0.111	.017	24
Yes	−0.372	<.001	−0.272	<.001	−0.100	.026	27

*Note.* PS=perceived stress; CS=compassion satisfaction; PDP=perceived disaster preparedness.

## Discussion

To the knowledge of the authors, this was the first study to elucidate the underlying mediating mechanisms among PS, PDP, and CS in hospital nurses by formulating and testing a mediation model. The findings support that the association between PS and CS is mediated by PDP and that this mediation effect is more prominent in nurses aged over 40 years, those working in hospitals in southern Taiwan, and those working rotating shifts.

Higher levels of PS leading to poorer CS outcomes in nurses have been widely reported in the literature (e.g., Kent et al., 2019; Ruiz-Fernández et al., 2020). Many interventions, including mindfulness-based stress-reduction programs (Alfurjani et al., 2024), mind-body-based interventions (Cepeda-Lopez et al., 2023), emotional-intelligence training (L. Mao et al., 2021), yoga (Bhardwaj et al., 2023), communication skill training (Saribudak & Ustun, 2024), and multimodal programs (El Khamali et al., 2018), have been recommended in the management of PS and its consequences and may help enhance CS in nurses. Moreover, receiving sufficient familial, social, or organizational support has been shown to reduce PS and enhance CS, highlighting the critical importance of cultivating supportive work environments to alleviate stress and promote professional fulfillment (Labrague & De Los Santos, 2020; Yehene et al., 2024).

The findings of this study further demonstrate CS and illustrate the necessity of interventions that target improvement in PS to enhance CS, as well as consider the levels of PDP in hospital nurses. Consistent with our hypothesis, PDP was identified as an important factor explaining why hospital nurses with higher levels of PS exhibit lower levels of CS. In this study, the participants exhibited a mean moderate perception of disaster preparedness, with more than half reporting no prior disaster training. Moreover, disaster preparedness perception was shown to be significantly linked to levels of empathy and psychological resilience, highlighting its critical influence on the professional capabilities of nurses (Ediz & Yanik, 2024). In addition, resilience and personal accomplishment have previously been shown to influence the quality of work life in nurses and contribute to sustaining their professional well-being (H.-F. Lee et al., 2024). Nursing is a stressful profession, especially during disasters such as the COVID-19 pandemic and while working in hospitals. Hospital nurses play an important role in preventing and managing epidemics such as COVID-19 (Tomaszewska et al., 2022). The mission of hospital nurses extends beyond promoting patients’ physical and mental health and improving the quality of medical care to encompass providing emergency services during disaster management and demonstrating agility, leadership, innovative problem-solving, and effective communication skills. Notably, adequate staffing and a supportive work environment are essential factors to enhancing the ability of nurses to perform these multifaceted roles effectively (L. Wang et al., 2025; Xue et al., 2020).

The results of this study confirm the mediating role of PDP by showing that the impact of PS on CS is fully mediated by PDP. In previous studies, the PDP level was shown to be positively associated with the CS level (M. H. Lee & Kim, 2018). This may be explained by nurses with higher PDP levels being able to provide better care, realize the meaning of nursing work, and feel happiness, all of which improve CS. Prior findings have shown disaster-related education and training and simulated emergency drills to be vital in effectively helping nurses understand how to deal with traumatic and complex situations in the work context, thus improving PDP levels to enhance CS (Jiang et al., 2022; Labrague et al., 2018; M. H. Lee & Kim, 2018; Songwathana & Timalsina, 2021). Therefore, intervention approaches that incorporate PDP should help improve CS levels in hospital nurses.

In this study, the mediation effect was shown to exert a stronger influence on older (>40 years) hospital nurses. Age has been previously correlated with PDP in nurses (Jiang et al., 2022; J. Wang et al., 2021). However, in this study, hospital nurses over 40 years old accounted for a significant proportion of the indirect effect of PDP on the relationship between PS and CS. The primary reason is that most older hospital nurses in Taiwan lack formal education in disaster nursing, resulting in potential gaps in their nursing knowledge and skills. Consequently, implementing on-the-job continuing education should particularly benefit older nurses, enabling them to enhance their abilities in disaster nursing.

Also, hospital nurses who worked in suburban areas in southern Taiwan required a higher level of PDP to reduce PS levels and improve CS. These findings are contrary to those of a previous study that reported hospital staffs working in urban areas had lower levels of CS than their peers working in rural areas during a global health pandemic (Kelly et al., 2022). Another study conducted on health care professionals showed levels of CS to be similar regardless of workplace location (Ruiz-Fernández et al., 2020). A significant association has been reported between hospital setting and PDP for explosive/incendiary accidents or attacks, with urban hospitals perceiving themselves as better prepared than their rural counterparts. This is because urban hospitals generally participate in more internal table-top exercises and full-scale simulations in collaboration with other organizations than rural hospitals (Vick et al., 2019).

Furthermore, nurses working rotating shifts were found to require a higher level of PDP to relieve PS and enhance CS. Shift work, especially day and night shifts, is a factor of influence on CS. Shift work is a fundamental element in continuity of care in nursing practice (Ruiz-Fernández et al., 2020). However, no definitive evidence has been found associating shift work with poorer psychological functioning in nurses. This association depends on several contextual and individual factors (Tahghighi et al., 2017). The findings of a prior study indicate that shift work may affect the physical, mental, and social dimensions of self-care and may attenuate the negative effects of this situation (Vidal-Blanco et al., 2019). In this study, nurses on a rotating shift schedule were found to be less likely to have prior experience with disaster nursing or related management training than those with fixed work schedules.

### Limitations

This study has several strengths and limitations. In terms of strengths, many participants were enrolled from hospitals across Taiwan. Thus, the results should be generalizable to nurses working in hospitals nationwide. Also, this study had a high completion rate, that is, 97.2%. In terms of limitations, the cross-sectional design did not allow changes over time to be examined. Thus, a longitudinal design may be adopted to examine changes in variable values over time. Also, the self-report questionnaire used may affect the reliability of responses. Future studies may use more objective measurement tools, for example, stress level monitoring, to improve the reliability of findings.

### Conclusions

PDP was identified as a resilience factor that helps nurses mitigate stress and sustain their CS during disaster events. Rather than merely a procedural component of disaster preparedness, PDP enhances adaptability and emotional endurance, allowing nurses to manage high-stress environments effectively. Strengthening resilience-building strategies within disaster preparedness programs, particularly for high-risk subgroups, is essential. In future research, researchers should explore how PDP fosters long-term psychological resilience, guides targeted interventions to enhance disaster preparedness, reduces stress, and sustains CS in nurses.

### Implications for Nursing Practice

The findings of this study have significant implications for nursing practice, particularly in the context of PDP and in terms of improving stress management efficacy among hospital nurses. The findings further emphasize the importance of recognizing and addressing the impact of PS on CS levels in nurses. Hospitals and health care institutions should implement interventions aimed at reducing stress levels in their nurses. Furthermore, the findings highlight the mediating role of PDP in the relationship between PS and CS. Therefore, interventions focused on enhancing PDP in nurses may effectively protect against the negative effects of PS on CS. Conducting training programs and simulation exercises, and facilitating access to resources aimed at enhancing nurse preparedness for disaster situations are valuable strategies to consider.
